# The effect of state and trait power on financial risk-taking: The mediating and moderating roles of optimism

**DOI:** 10.1371/journal.pone.0276878

**Published:** 2022-10-31

**Authors:** Katarzyna Sekścińska, Joanna Rudzinska-Wojciechowska, Diana Jaworska

**Affiliations:** 1 Faculty of Psychology, University of Warsaw, Warsaw, Poland; 2 Centre for Economic Psychology and Decision Sciences, Kozminski University, Warsaw, Poland; University of Engineering and Technology, PAKISTAN

## Abstract

The research aimed to further develop knowledge on the mechanisms that enhance risk-taking propensities among powerful people. Three studies (*N*1 = 328, *N*2 = 388, *N*3 = 267) investigated the role of optimism in the relationship between sense of power and financial risk-taking, controlling for the state of power. Study 1, correlational, analyzed whether the relationship between sense of power and risky financial choices is serially mediated by general optimism and financial risk perception. The results confirmed the initial hypotheses. The second, experimental, study investigated the role of states of power and lack of power in explaining people’s financial decisions as well as their influence on people’s situational optimism and perception of risk. The results indicated that people in a state of power differed from people lacking power in terms of their situational optimism and the riskiness of their financial choices; however, they did not differ in terms of risk perception. People having power were more optimistic, invested more, and made riskier gambling choices than those in control conditions and those who lacked power. The third, experimental, study investigated the single and joint moderating effects of the states of optimism and power in explaining the positive relationship between sense of power and risky investing and gambling choices. In line with our expectations, the results of the study showed that the states of power and optimism jointly moderated the positive relationship between the sense of power and risky financial choices. This effect was the strongest under the state of power and optimism conditions and the weakest when lack of power and pessimism were induced; thus boundary conditions for previously observed mechanisms were identified. The results of the research shed light on the way optimism mediates and moderates the effect of power on financial risk-taking and thus contributes to theoretical knowledge of the consequences of power.

## 1. Introduction

Financial choices made by powerful people might have a large impact on the well-being of other people (e.g., family members, employees, and citizens), and the consequences of such choices might be even greater when they involve risks. Meanwhile, numerous studies have confirmed that power alters the way people process information and make decisions (see [1] for review). Among the many consequences of power, it is well-documented that power facilitates risk-taking in many aspects of life, financial risk-taking being no exception [1–4]. Such risks can have severe consequences for the financial security of one’s household, firm, or community. Although a better understanding of the mechanisms that enhance risk-taking propensities among powerful people might help them to make better informed financial decisions, this issue is severely understudied. According to our best knowledge, there are no previous studies that investigate the mechanisms standing behind the link between power and risky financial choices. At the same time, exploring this link could help us understand the mediators and boundary conditions of this relationship.

Previous studies have established that optimism moderates the link between power and risk-taking [5] and demonstrated that people with a greater sense of power have more optimistic perceptions of their own future and the future in general, and that an optimistic perception of risky behaviors mediates preferences for risk-taking. However, there is the question as to whether the relationship between power and risky choices is mediated not only by a more optimistic perception of risk but also by optimism per se. The role of risk perception is also unclear. We propose that greater sense of power translates into greater optimism, which in turn leads to lower risk perception and results in riskier financial choices and our purpose is to test this serial mediation in our research project. Moreover, little is known about factors that mitigate and exaggerate the effects of power on risk-taking. In the search for boundary conditions for this relationship, we take into account that even people with high sense of power might find themselves under power of someone else and the reverse is also true: people low in sense of power might gain situational power over others. Moreover, powerful people might sometimes find themselves in circumstances that temporarily make them pessimistic and, conversely, optimism may be induced in those lacking power. Therefore, the objective of the current research is to investigate whether state of power and state of optimism jointly impact the relationship between sense of power and financial risk-taking.

In this research, we observe the effects in two domains of risky financial choices: investment and gambling. Examining multiple forms of financial risk-taking allows for enhancing confidence in the results. Moreover, we both measure and manipulate optimism. We also consider power both as a trait and as a state and take into account possible interactions between them.

### 1.1. Power

Power is often defined as asymmetric control over valued resources in social relations [6]. This definition captures the relative state of dependence between one or more parties [1, 6, 7]. It is important to stress, that the nature of power is subjective and dynamic across situations and contexts: e.g. a manager might have power over employees in terms of their career advancement but at the same the employees have power over the employer at the time they start thinking about quitting [1]. Feeling powerful or powerless can be activated by many factors, both structural (e.g. assigning individuals to a hierarchal role of a boss/employee for a duration of a task), or cognitive (e.g., asking participants to write about a time they had or lacked power or to unscramble sentences that contain words related to having or lacking power) (see [8] for a review). Thus, it is possible to manipulate the subjective sense of how powerful one feels at a given moment.

In addition to situational factors, there are also individual differences associated with power. In this sense, power is often understood as a perception of one’s ability to influence others and as such, it is considered an individual variable (sense of power, SOP) [9]. Cues to the possession of power create a sense of power, which in turn has been shown to influence the way people process information, make decisions, and take actions (e.g., [5, 10, 11]; see [1] for further review of the psychology of power and consequences of power).

### 1.2. Power and risky financial decisions

Power can affect many aspects of people’s functioning, and decisions made in a financial context are no exception. For example, it has been shown that power influences the way people spend money: low power leads to a preference for high-status objects and conspicuous consumption, whereas high power leads to a focus on the functional value offered by products and rejection of conspicuous consumption [12]. More powerful people are also less likely than those with less power to spend all their money on current consumption: they often decide to put some money aside for their future needs [13, 14].

In the context of risky financial decisions, it is important to note that greater power facilitates risk-taking behaviors in various domains, such as negotiations [5, 15], engaging in unprotected sex [5], marital infidelity [16], and risky food consumption [17]. Several studies have also documented that power increases the propensity to take financial risks. On a corporate level, the power held by a CEO is positively related to excessive and unmanaged risk-taking by a firm [2, 18, 19]. When it comes to decisions made by individuals, studies show that people in a state of power are more likely to take gambling risks [1]. A link between greater levels of power measured with the Sense of Power Scale [9] and riskier gambling choices has also been observed [20, 21]. Similarly, experimentally-induced state of having power results in greater investment risks than low power [3, 20].

The link between power and the propensity to take financial risks is supported by several studies. However, the mechanisms of the positive effect of power on risk-taking still need to be investigated to understand boundary conditions for this relationship. Currently, the potential roles of variables that may mediate and moderate this relationship are not well understood. Explanations of this effect often focus on the way power alters one’s perception of risk and, consequently, impacts decisions and actions. So far it has been established that power impacts the way people perceive the potential consequences of their choices. Specifically, power makes failure seem less probable and painful, and thus decreases the level of the perceived risk of action [11, 22, 23]. Moreover, it has been shown that more powerful individuals are more sensitive to potential gains than people with less power [10, 24, 25]. Power also alters the way one perceives risk–it increases overconfidence [26], perception of control over outcomes [27], and optimism [5, 26]. Regarding optimism, it has been demonstrated that people with a greater sense of power have a more optimistic perception of their own future and future in general and that optimistic perception of risky behaviors mediates preferences for risk-taking [5]. In this paper, we aim to further develop knowledge on the mediating and moderating roles of optimism in the relationship between power and risky financial choices.

### 1.3 The interplay of state and trait power

As explored above, power can be considered both as an individual difference (trait–sense of power; SOP) variable and as a psychological state. Overall, research measuring people’s sense of power has tended to yield findings consistent with manipulations of power (see [8] for a review). However, state power and trait power have seldom been considered together in single experiments. Nevertheless, there is some evidence that these might interact, although the pattern of interactions between SOP and power manifested as a state is not straightforward. For instance, Chen, Langner, and Mendoza-Denton [28] demonstrated that in a situation where a person’s SOP and experimentally-assigned power matched, regardless of whether this match involved high or low levels of both types of power, people’s behaviors were more congruent with their self-reported emotions and traits than when trait and state power were not matched. Another study indicated that exposure to situational power (relative to lack of power or a neutral context) increases vengeance among people with a low level of dispositional power and reduces it among people with a high level of dispositional power [29]. In the context of risky financial choices, one study demonstrated that SOP is positively related to the riskiness of investment portfolios and gambling choices. A similar pattern was observed when a state of power/powerlessness was situationally induced: participants in high-power conditions took greater investment and gambling risks than did those in low-power conditions. Importantly, however, there was an interaction between trait and state power. A positive relationship between SOP and the propensity to take financial risks occurred only in the high-power state condition, while there was no such relationship in the low-power state condition [20]. In conclusion, the interaction effect of trait and state power is not well understood, nor are its effects on optimism and risk perception in risky financial decision-making.

### 1.4. Optimism and financial risk-taking

Optimism reflects the extent to which people hold generalized favorable expectancies for their future [30]. Optimists expect good things to happen to them, whereas pessimists expect bad things [30]. Although optimism is commonly considered a trait, it is possible to train an individual to be optimistic [31]. Thus, optimism has both trait and state components, and researchers have demonstrated the effectiveness of optimism interventions (see [32]) and designed measures for state optimism (e.g., [33]). Individual differences in optimism are relevant in various aspects of life and, overall, optimism is beneficial (for a review, see [34]). However, there is a potential drawback to optimism: it can induce a greater tendency to take risks based on positive expectations about the future [35]. Studies have shown that unrealistic optimism is a predictor of risk perception (e.g., [36]).

In the domain of risky financial choices, the link between optimism and risky investment choices is well established and the results of studies are consistent. In general, optimists are more likely to invest money than people low in optimism. Specifically, they are more likely to own stocks [36] and they tend to allocate a larger fraction of their equity wealth to individual stocks [37–39]. Moreover, optimistic investors choose risky portfolios over risk-free portfolios for their investments [40]. Similarly, studies on gambling indicate that optimists have greater positive expectations for gambling than pessimists and are less likely to reduce their betting after losses [41].

### 1.5. Risk perception and financial risk-taking

When discussing risky decision-making, it is important to explore the issue of risk perception. This psychological construct is defined as the “subjective assessment of the probability of a specified type of accident happening and how concerned we are with the consequences” [42]. People’s perceptions of the riskiness of choice alternatives often diverge from conventional indicators of risk, such as the probability of loss or standard deviation of possible outcomes [43]. Moreover, the way people subjectively perceive a given level of risk can differ significantly from individual to individual and also depends on situational factors [44]. Importantly, risk perception is often a better predictor of risk-taking behaviors than objective measures of risk [45, 46]. Research shows that risk perception mediates risky choices [43]. High-risk perception is negatively related to risk-taking-behavior [47] and the domain of risky financial choices is no exception [46]. This was demonstrated in the case of individual investors [48], as well as in the case of finance professionals [49]. Perceived risk also predicts risky gambling choices [45].

### 1.6. Current studies

Although the link between power and the propensity to take risks is well established [5, 15–17], also in the domain of financial risk-taking, with a few studying demonstrating that greater power is linked to a greater propensity to gamble and invest money, both private and corporate [1–4, 18, 19], potential mediators and moderators of such relationship are still understudied, as are boundary conditions to the effects. One of the potential variables that might explain this relationship is optimism. Studies show that power increases optimism [5, 26]. It was also demonstrated that optimism induces a greater tendency to take risks and lowers risk perception [35]. Risk perception in turn was demonstrated to be negatively related to risk-taking-behavior [46–49].

We are aware of only one exploration to date that investigates the mediating role of optimism in the relation between power and risk-taking. In two studies, Anderson and Galinsky [5] demonstrated that participants in a high-power condition were more willing to engage in risky behavior (casual sex without a condom) and that this was due to a more optimistic perception of the risks involved than people with low power (study 4). Moreover, in the context of a dyadic negotiation (study 5), they showed that the link between SOP and risk-taking behavior (in this case, risk in sharing information) was partially mediated by perceptions of risk (in this case, optimistic perception of the negotiation situation). These findings shed light on the mechanisms underlying the link between power and risk-taking and indicate the importance of the mediating role of optimism. However, it is important to establish whether the relationship between power and risky choices is mediated not only by a more optimistic perception of risk but also by optimism per se. Moreover, the moderating role of optimism needs to be investigated in the search for factors mitigating and exaggerating the effects of power on risk-taking. Finally, it should be tested whether the state of power changes the role of optimism in the relationship between SOP and risk taking.

In this paper, we present three studies examining the mediating and moderating roles of optimism in the relationship between power and risky financial choices in two domains: investment and gambling. The first study investigated whether the relationship between SOP and risky financial decisions is mediated by participants’ general optimism level and risk perception. In this study, participants made incentivized investment and gambling decisions and assessed the subjective level of risk they took while making them. Next, we assessed participants’ levels of SOP and general optimism. The second study took into account the results of previous studies demonstrating that SOP interacts with state of power in the process of making risky financial choices. It also builds upon the results of Study 1, which indicated the mediating role of optimism and risk perception in the relationship between power and risky financial choices. Therefore, Study 2 examined whether states of power and lack of power impact situational optimism and risk perception. In this study, we manipulated participants’ levels of power and measured the levels of optimism they felt at that moment. Finally, participants made incentivized investment and gambling choices and assessed the subjective level of risk they took while making them. Study 3 aimed to verify whether the role of sense of power in explaining risky financial decisions in states of having or lacking power is different depending on state of optimism. We manipulated participants’ levels of power and optimism. We also observed their choices in incentivized investment and gambling tasks.

To establish appropriate sample sizes for the studies, a priori power analyses were conducted using G*Power [50, 51], with α = .05 and 0.80 power. For all studies, we aimed to recruit 15% more participants than indicated by the results of the G*Power analyses, because in most of the previous studies using Holt and Laury’s lottery task, on average 15% of the sample had to be excluded from the analyses because of multiple switching points and/or choosing dominated options in this task.

## 2. Study 1: Power and risky financial choices–the mediating roles of optimism and risk perception

### 2.1. Study 1 aim

**Fig 1:**
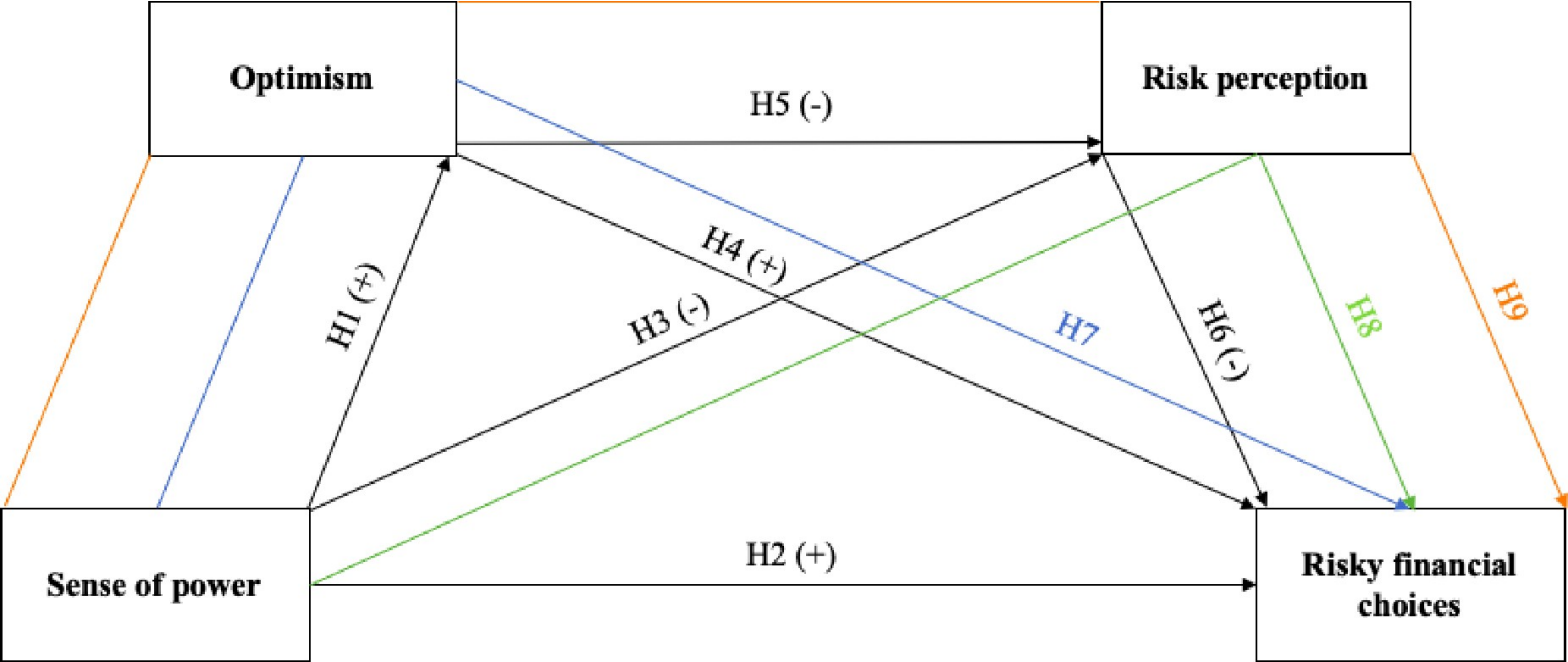
Study 1 framework and hypotheses.

Study 1 analyzed whether the relationship between SOP and risky financial choices in investing and gambling domains is serially mediated by general optimism and financial risk perception. Based on previous research, we expected that SOP would be positively related to optimism and risky financial choices and negatively related to risk perception (H1, H2, H3). Moreover, we expected that optimism would be positively related to risky financial choices (H4) and negatively related to risk perception (H5). We also hypothesized that risk perception would be negatively linked to risky financial choices (H6). Given the aforementioned relationships, we hypothesized the existence of a serial mediating path from SOP to risky financial choices via optimism and risk perception. The following hypotheses were investigated: the relationship between SOP and risky financial choices would be mediated by optimism (H7) and risk perception (H8), and would be serially mediated by optimism and risk perception (H9). The research framework and hypotheses are presented in Fig 1, with the hypothesis number being placed above the corresponding arrow. The study was preregistered at: https://osf.io/nuqj9/?view_only=c38b2a6c37d34918a5e183c3c7c86b72 (DOI 10.17605/OSF.IO/NUQJ9).

### 2.2. Method

#### 2.2.1. Participants

Data were collected from 328 Polish working adults (166 female and 162 male; aged 19–64 years, *M* = 40.88 years, *SD* = 12.55).

The studies were conducted with the CAWI methodology using the online Polish ARIADNA participant panel, which has over 110,000 active adult panel members. Email invitations were sent to potential participants, diverse in terms of age, gender, and level of education. Each email contained a unique link to the study that worked only once and only for the particular panel member. When the participant clicked on the link, they were transferred to ARIADNA’s research platform and began the study. Participants who took part in the first study were not invited to the next studies and were therefore unable to take part in them. Respondents were awarded points for participating that they could later exchange for rewards from a pool of several hundred products offered by the platform running the panel. Additionally, extra points were awarded to participants depending on their choices during the investment and gambling tasks.

*2*.*2*.*1*.*1*. *Ethics approval and informed consent statements*. The Ethics Board at Faculty of Psychology, Warsaw University approved the study. All procedures performed in studies involving human participants were in accordance with the ethical standards of the institutional research committee and with the 1964 Helsinki Declaration and its later amendments or comparable ethical standards. Participants written informed consent was collected in the online Polish ARIADNA participant panel.

#### 2.2.2. Materials and procedure

*2*.*2*.*2*.*1*. *Sense of power*. The generalized Sense of Power Scale [9] was used to assess participants’ sense of power. Participants rated their agreement with eight statements such as “In my relationships with others I can get others to do what I want” on a scale from 1 (*definitely disagree*) to 7 (*definitely agree*). Four items were reverse coded, and responses were averaged to create an indicator of each participant’s sense of power.

***2*.*2*.*2*.*1*.*1*. *Financial risk-taking (gambling subdomain)*.** The lottery choice task proposed by Holt and Laury [52] was used to assess participants’ propensity to take risks in the domain of gambling. Participants made ten choices between paired lotteries (Lottery A and Lottery B). The probability of the high-payoff outcome increased in both lotteries, starting with *p* = 0.1 and ending with *p* = 1. However, in each pair, Lottery B was the riskier option because the potential payoffs for Lottery A were less variable than for Lottery B. Hence, the index of risky lottery choices was reflected by the sum of Lottery B choices. This task was incentivized: at the end of the study one of the lotteries was selected at random and the result of the lottery was determined by a virtual throw of a 10-sided die. Note that in this task a rational decision-maker should neither choose option A in the last decision nor have multiple switching points between lotteries A and B. Hence, such participants were excluded from the analyses (see [53]). See S1 File for the exact wording of the task.

***2*.*2*.*2*.*1*.*2*. *Risk perception (gambling subdomain)*.** After completing the lottery choice task, participants were again presented with decisions 1–3 and asked to recall and describe on a scale of 0 (risk-free) to 100 (extremely risky) how risky it seemed to them to choose option B (meaning to give up option A) while they were making each of these decisions.

***2*.*2*.*2*.*1*.*3*. *Financial risk-taking (investment subdomain)*.** An incentivized tool that measures the riskiness of investment choices was used. It consisted of two investment decisions concerning PLN 10,000 (~ USD 2,400) each. Participants split this amount between an interest-free bank account and investments in stocks. At the beginning of each decision, a chart showing changes in the price of a hypothetical stock over 12 months was presented. Participants were informed that the chart and how it continued to change were computer-generated. After that, the participants were asked to make their first decision. Then they were shown another chart and made a second investment decision. At the end of the study, the participants received feedback about changes in the stock price and their final balance. If a participant decided to assign all the money to the interest-free bank account in all the decisions, they would receive 4 extra points. However, if the participant decided to invest in stocks, they would receive between 1 and 7 extra points. Investment riskiness was calculated as the percentage of the amount of money assigned to stocks in each decision.

***2*.*2*.*2*.*1*.*4*. *Risk perception (investment subdomain)*.** After completing both of the investment task decisions, the participants were again presented with the two charts depicting share prices and asked to reflect on how they perceived the risk of investing in these shares. Participants assessed the riskiness of investment separately for decision 1 and decision 2 on a scale of 0 (risk-free) to 100 (extremely risky).

**General optimism** was measured with the Polish version of the 9-item Optimism Scale [54]. The scale consists of such items as: "I see the positive side of things" or "I am confident I can overcome problems". Participants rate their agreement with the statements on a scale from 1 *(strongly disagree*) to 5 *(strongly agree*). The responses were averaged to create an indicator of general optimism (one item was reverse coded). See Supplementary Materials S1 File for details on the Polish adaptation.

**Fig 2:**
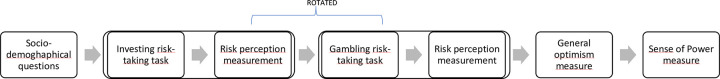
Flowchart of Study 1 procedure.

*2*.*2*.*2*.*2*. *Procedure*. Participants completed the battery of research tools in the following order: socio-demographic questions, investing risk-taking task with related questions measuring risk perception, and gambling risk-taking task with related questions measuring risk perception (investment and gambling tasks presented in rotated order), general optimism scale, and the sense of power scale. The flowchart of the study is presented in Fig 2.

### 2.3. Results

#### 2.3.1. Correlations between variables

As shown in Table 1, all the expected relationships between variables were observed. SOP was positively and moderately related to optimism and positively albeit weak related to risky investment choices (decision 1 and decision 2) and risky gambling choices. Moreover, SOP was weakly and negatively related to investment (decision 1 and decision 2) and gambling risk perception. Optimism was positively albeit weakly related to risky investment choices (decision 1 and decision 2) and gambling choices. What is more, optimism was also weakly and negatively related to investment (decision 1 and decision 2) and gambling risk perception. The relationship between the two investment choices was positive and strong. Moreover, the correlation between risk perception of investment decision 1 and 2 was also strong and positive.

**Table 1:** Means, standard deviations, and Pearson correlations between variables.

	*M*	*SD*	2	3	4	5	6	7	8
1. Sense of power	4.20	0.81	.43**	.23**	.18**	-.24**	-.22**	.15**	-.22**
2. Optimism	3.44	0.62		.26**	.26**	-.21**	-.21**	.24**	-.23**
3. Risky investment choices–decision 1	48.82%	29.64%			.77**	-.36**	-.32**	.18**	-.31**
4. Risky investment choices–decision 2	49.53%	29.19%				-.29**	-.39**	15**	-.29**
5. Investment risks perception–decision 1	57.91	20.92					.73**	-.13*	.35**
6. Investment risks perception–decision 2	57.95	21.47						-.11^t^	.33**
7. Risky gambling choices	4.61	2.52							-.24**
8. Gambling risk perception	67.04	22.47							

* *p* < .05

** *p* < .01

*Notes*: analyses for variables 7 and 8 were conducted on a reduced sample (*n* = 324; 4 participants who made dominated choices in the tenth decision in the lottery task were excluded from the analyses); correlations related to the same model remained significant (*p* < .05) after Bonferroni correction for multiple comparisons, datasource: https://osf.io/nuqj9/?view_only=c38b2a6c37d34918a5e183c3c7c86b72 (DOI 10.17605/OSF.IO/NUQJ9); research method: correlational.

Four participants were excluded from all the analyses related to the risky gambling choices due to their choice of option A in the last choice in the lottery task, which is a dominated option and should not be chosen by a rational decision-maker [53].

#### 2.3.2. Mediating effect of optimism and risk perception on the relationship between sense of power and risky financial choices

The PROCESS macro (Model 6) developed by Hayes [55] was used to determine the serial mediating effect of optimism and risk perception in the relationship between sense of power and risky financial choices (in both investing and gambling subdomains). PROCESS with the bootstrapping method was used to generate a bias-corrected bootstrap confidence interval (CI) based on 5000 resamples from the data.

*2*.*3*.*2*.*1*. *Risky investment choices*. We conducted two serial mediation analyses for risky investment choices with the first and second decisions as outcome variables. As the obtained results were very similar, we present the analyses for decision 1 in the main text, while the analyses for decision 2 are presented in the S1 File.

**Fig 3:**
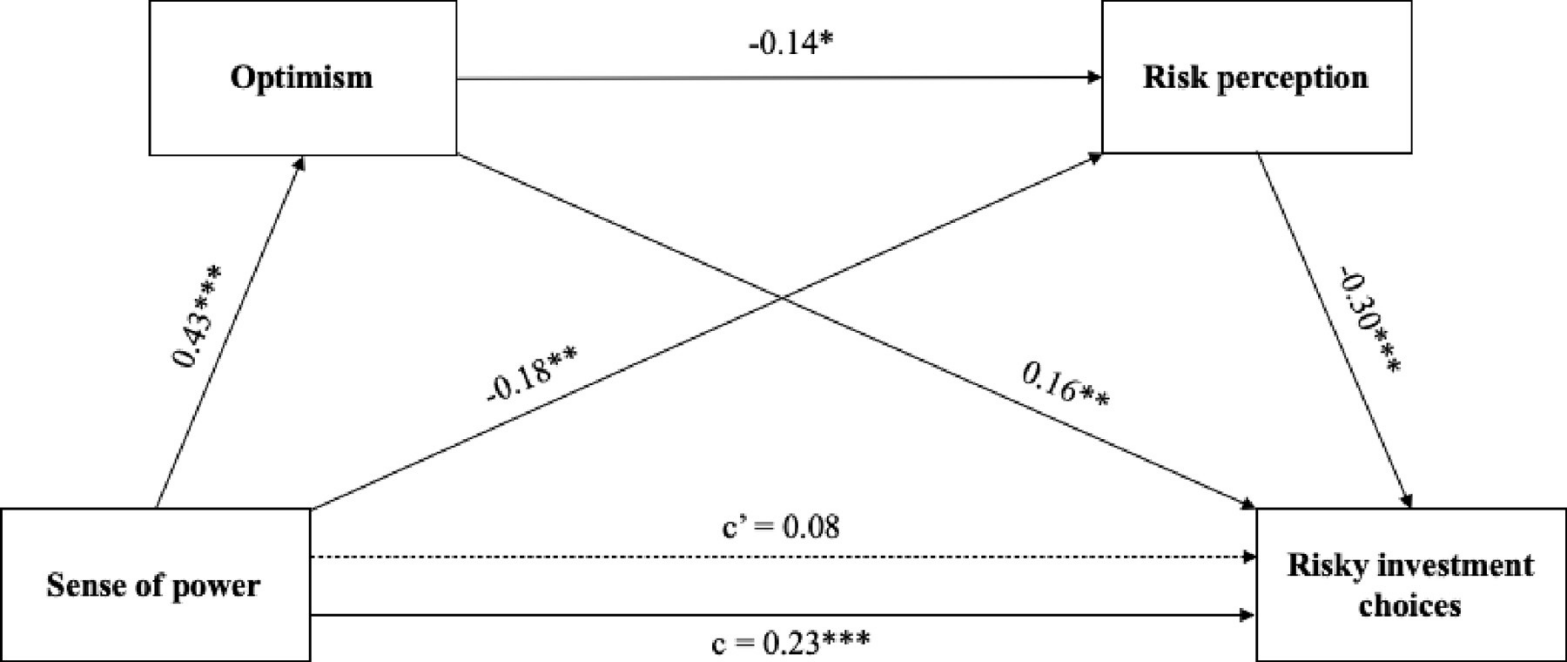
Serial multiple mediation model explaining risky investment choices. For the path directly linking sense of power and risky choices, the direct effect is shown above the upper arrow, and the total effect (the effect without controlling for the mediator variable) is shown below the lower arrow. The statistics represent standardized effects. Solid arrows represent significant effects (***p < .001, **p < .01, *p < .05**; c–total effect, c’–direct effect).

As presented in Fig 3, in line with our predictions SOP was positively related to optimism and risky investment choices (which supports H1 and H2) and negatively related to risk perception (which supports H3). Moreover, optimism was positively related to risky financial choices, and negatively related to risk perception (supporting H4 and H5, respectively). Furthermore, in line with H6, risk perception was negatively linked with risky financial choices. Finally, the direct effect (c’) of SOP on risky investment choices was not significant.

The overall regression model explaining risky investment choices with SOP, optimism, and risk perception introduced as independent variables was significant (*R*^*2*^ = .17; *F*(3,324) = 21.82*; p <* .001). As predicted in H1, the total effect (c) of SOP on risky investment choices was positive and significant. The direct effect (c’) of SOP on risky investment choices was not significant in the serial mediation model, while the total indirect effect was significant (c–c’ = .14; BootSE = .03; 95% CI = .08 to .20), which indicates full mediation. Further analyses showed that the total indirect effect of SOP on risky investment choices can be decomposed into three different significant partial indirect effects. The first of them is the effect via optimism. SOP was related to increased optimism, which translated into risky investment choices (partial effect 1, indirect effect = 0.07; BootSE = .03; 95% CI = .02 to .12). The second partial indirect effect was via risk perception–SOP was related to lowered risk perception which in turn was related to riskier investment choices (partial effect 2, indirect effect = 0.06^2^; BootSE = .03; 95% CI = .01 to .11) Finally, a serial mediation effect was also observed. SOP translated into greater optimism, which led to lower investment risk perception, which in turn led to riskier investment choices (partial effect 3, indirect effect = 0.02^2^; BootSE = .01; 95% CI = .01 to .04). Among the three partial indirect effects, 1 and 2 played similar roles, while the role of partial indirect effect 3 was smaller.

**Fig 4:**
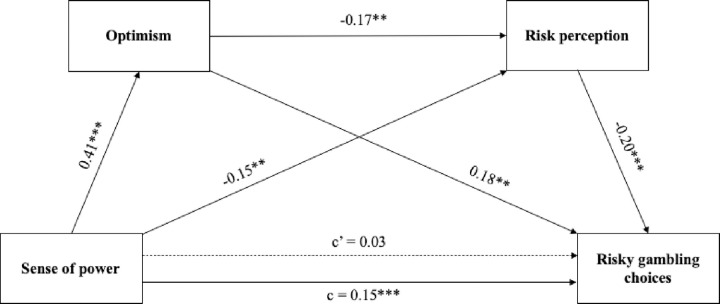
Serial multiple mediation model explaining risky gambling choices. For the path directly linking SOP and risky choices, the direct effect is shown above the upper arrow, and the total effect (the effect without controlling for the mediator variable) is shown below the lower arrow. The statistics represent standardized effects. Solid arrows represent significant effects (***p < .001, **p < .01, *p < .05**; c–total effect, c’–direct effect).

*2*.*3*.*2*.*2*. *Risky gambling choices*. The serial multiple mediation model, presented in Fig 4, showed that SOP was positively related to optimism and risky gambling choices (which supports H1, H2) and negatively related to risk perception (which supports H3). In line with H4 and H5, optimism was positively related to risky gambling choices and negatively related to risk perception. Risk perception was negatively linked to risky gambling choices, which supports H6. Finally, the direct effect (c’) of SOP on risky gambling choices was not significant.

The overall regression model explaining risky gambling choices with SOP, optimism, and risk perception as independent variables was significant (*R*^*2*^ = .10; *F*(3,320) = 11.25*; p <* .001). As predicted in H1, the total effect (c) of SOP on risky gambling choices was positive and significant. The direct effect (c’) of SOP in risky gambling choices was not significant in the serial mediation model, while the total indirect effect was significant (c-c’ = .12; BootSE = .03; 95% CI = .06 to .18), which indicates full mediation.

Then the obtained total indirect effect of SOP on risky gambling choices was decomposed into three different partial indirect effects. All of them were significant. The first one was the effect via optimism. SOP was related to increased optimism, which translated into risky gambling choices (partial effect 1, indirect effect = .08; BootSE = .03; 95% CI = .03 to .13). The second partial indirect effect was via risk perception–SOP was related to lowered risk perception which in turn was related to riskier gambling choices (partial effect 2, indirect effect = 0.03; BootSE = .02; 95% CI = .003 to .06). Finally, the serial mediation effect was also observed. SOP translated into greater optimism, which led to lower investment risk perception, which led in turn to riskier gambling choices (partial effect 3, indirect effect = 0.01; BootSE = .01; 95% CI = .002 to .03). Among the three partial indirect effects, effect 1 seemed to be the most important, while the roles of effects 2 and 3 were smaller.

## 3. Study 2: State of power, optimism, risk perception, and risky financial choice

### 3.1. Study 2 aim

Study 1 showed that the relationship between sense of power and risky financial choices is mediated by optimism and risk perception. However, power may be situationally changed. Consequently, the state of power may influence situational optimism and risk perception, as well as risky financial decisions. Study 2 aimed to analyze the role of the states of having and lacking power in explaining people’s financial decisions, situational optimism, and perception of risk.

Based on previous research, we expected that a state of power would increase the riskiness of financial decisions, while a state of lack of power would lower it (H10). We also predicted that a state of power would increase situational optimism, while the effect of a state of lack of power would be the opposite (H11). Moreover, we expected that a state of power would lower risk perception, while a state of lack of power would increase it (H12). The study was preregistered at: https://osf.io/pf4h3/?view_only=9e00a6247bb74f0793999abf609e835c (DOI 10.17605/OSF.IO/PF4H3).

### 3.2. Methods

#### 3.2.1. Participants

Data were collected from 388 Polish working adults (217 female and 171 male; aged 19–72 years, *M* = 41.63 years, *SD* = 13.74). The Study was conducted using the same research panel as Study 1 and the participants were also recruited analogously.

*3*.*2*.*1*.*1*. *Ethics approval and informed consent statements*. The Ethics Board at Faculty of Psychology, Warsaw University approved the study. All procedures performed in studies involving human participants were in accordance with the ethical standards of the institutional research committee and with the 1964 Helsinki Declaration and its later amendments or comparable ethical standards. Participants written informed consent was collected in the online Polish ARIADNA participant panel.

#### 3.2.2. Materials and procedure

*3*.*2*.*2*.*1*. *Power as a state*: *Experimental manipulation*. States of having power or lacking power were induced by putting participants in positions in which they either evaluated and rewarded other people’s work (state of power) or were subjects of such an evaluation. At the beginning of the procedure, all participants were informed that a creative task was being conducted among participants of the research panel they belonged to. They were also informed that the creative task involved participants writing three valid sentences in which they had to use three provided words in such a way that it was difficult to guess which word had been provided, and that the participant’s performance on the task would be rewarded with extra points based on evaluation. Participants in the **state of having power group** were asked to evaluate the performance of another panelist on this task and to decide whether to award this panelist with extra points. Participants in the **state of lacking power group** were informed that they would be asked to perform a creative task at the end of the study and that another panelist would be asked to evaluate their performance and decide whether to reward them. Then they were presented with the same material that participants from the state of having power group evaluated (ostensibly so that they could understand the task better). At the end of the procedure, these participants were asked to write their own three sentences. In the **control condition,** participants were presented with the same material the other groups saw and then asked to perform a creative task that would be transferred to a database. The exact wording of the experimental manipulation can be found in the S1 File, together with the results of the pilot study that tested the effectiveness of this procedure.

**Situational general optimism** was measured using the same Polish version of the Optimism scale [54] as in Study 1, but with modified instructions. Instead of asking participants “what you usually think and feel” we asked them: “what you are thinking and feeling at this moment” to capture their current situational level of general optimism. Participants rated their agreement with each of the statements on a scale from 1 (*strongly disagree*) to 5 (*strongly agree*). The responses were averaged to create an indicator of situational general optimism score (one item was reverse coded). The reliability of the tool was good in our sample (Cronbach’s *α* = .92).

**Risk perception and financial risk-taking in the gambling and investing subdomains** were measured as in Study 1.

**Fig 5:**
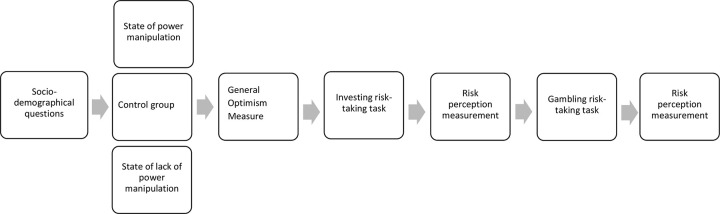
Flowchart of Study 2 procedure.

*3*.*2*.*2*.*2*. *Procedure*. At the beginning of the study, after completing socio-demographical questions, participants were randomly assigned to one of three groups: the state of power experimental group (*n*_1_ = 132), the state of lack of power experimental group (*n*_2_ = 133), or the control group (*n*_3_ = 123). Then the participants took part in the experimental task and, after doing so, their Situational General Optimism was measured. Next, they made investment and lottery choices and their level of risk-perception was assessed. Finally, the participants were informed about the number of extra points they had earned and fully debriefed. The flowchart of the study is presented in Fig 5.

### 3.3. Results

Seventeen participants were excluded from the analyses based on the same criteria as in Study 1 [53]. A sensitivity analysis using G*Power [50] indicated that, given α = 0.05 and an assumed power of 0.80, a sample size of 371 participants would be sufficient to detect a small effect (ƒ^2^ (V) = 0.025) in a MANOVA model with 3 groups and 7 response variables. Databases are available at: https://osf.io/pf4h3/?view_only=9e00a6247bb74f0793999abf609e835c.

#### 3.3.1. Descriptive statistics

Descriptive statistics for the total analyzed sample (*N* = 371) as well as for each experimental group (*n*_1_ = 117; *n*_2_ = 132; *n*_3_ = 122) are presented in Table 2.

**Table 2:** Descriptive statistics.

	Total		State of power group		State of lack of power group		Control group	
	*M*	*SD*	*M*	*SD*	*M*	*SD*	*M*	*SD*
1. Situational Optimism	3.59	0.77	3.88	0.65	3.34	0.86	3.58	0.68
2. Risky investment choices–decision 1	49.51%	29.28%	59.28%	28.05%	40.43%	29.51%	49.99%	27.25%
3. Risky investment choices–decision 2	48.43%	29.98%	58.70%	28.36%	38.94%	29.50%	48.85%	28.89%
4. Investment risks perception–decision 1	59.78	18.27	60.38	17.63	61.66	16.56	57.18	20.37
5. Investment risks perception–decision 2	59.71	18.77	58.10	19.14	62.08	17.51	58.69	19.61
6. Risky gambling choices	5.31	2.42	6.43	2.06	4.39	2.56	5.24	2.14
7. Gambling risk perception	69.96	19.26	71.99	17.10	70.89	17.37	67.01	22.66

* *p* < .05

** *p* < .01

*Note*: analyses for variables 6 and 7 were conducted on a reduced sample (*n* = 371), as 17 participants who made irrational choices in the lottery task were excluded from the analyses; datasource: https://osf.io/pf4h3/?view_only=9e00a6247bb74f0793999abf609e835c (DOI 10.17605/OSF.IO/PF4H3); research method: experimental.

#### 3.3.2. State of (lack of) power, optimism, risk perception, and risky financial choices

To analyze the role of states of power and lack of power on people’s optimism, financial risk perception, and risky financial choices in both investing and gambling domains, a MANOVA analysis was conducted with state of power (power, lack of power, control) as independent variables, and risky investment choices, investment risk perception, risky gambling choices, and gambling risk perception as dependent variables (DVs).

The results of the MANOVA showed that there was a statistically significant difference in the DVs based on the state of power, *F* (14, 724) = 7.48, *p* < .001; Wilk’s Λ = 0.77, partial *η*^*2*^ = .13. Further analyses showed that state of power had a statistically significant effect on situational optimism, (*F* (2, 368) = 16.60; *p* < .001; partial *η*^*2*^ = .08), both risky investment choices (decision 1: *F* (2, 368) = 13.78; *p* < .001; partial *η*^*2*^ = .07; decision 2: *F* (2, 368) = 14.47; *p* < .001; partial *η*^*2*^ = .07), and risky gambling choices (*F* (2, 368) = 24.88; *p* < .001; partial *η*^*2*^ = .12). All the effects observed remained significant after Bonferroni correction for multiple ANOVAs. There were no significant differences between state of power groups in their investment risk perception (investment decision 1: *F* (2, 368) = 2.01; *p* = .14; partial *η*^*2*^ = .01; investment decision 2: *F* (2, 368) = 1.66; *p* = .19; partial *η*^*2*^ = .01) and gambling risk perception (*F* (2, 368) = 2.25; *p* = .11; partial *η*^*2*^ = .01).

We then followed up the significant ANOVAs with Tukey’s HSD post-hoc tests. Situational optimism was statistically greater in the state of power group than in the state of lack of power *(p* < .001) and control (*p* = .006) groups. Moreover, the control group was more optimistic than the lack of power group (*p* = .03).

In both decision 1 and decision 2, the amount of money invested in stocks was greater in the state of power group than in the state of lack of power group (*p*s < .001) and control group (decision 1: *p* = .04; decision 2: *p* = .03). Moreover, in both decisions, participants in the state of lack of power group assigned less money to stocks than did the control group (*p*s = .02).

In terms of gambling choices, the state of power group made riskier choices than both the state of lack of power and the control group (*p*s < .001). Furthermore, participants in the state of lack of power group made less risky gambling choices than did the control group (*p* = .01).

## 4. Study 3: Sense of power and risky financial choices–a moderated moderation model of optimism and state of power

### 4.1. Study 3 aim

Taking into account the results of previous studies indicating that state of power interacts with sense of power and based on the results of Study 2, which demonstrated that state of power and lack thereof impacts levels of optimism and risky financial decisions, Study 3 aimed to test the single and joint moderating effects of state of optimism and state of power in explaining the positive relationship between sense of power and risky investing and gambling choices.

**Fig 6:**
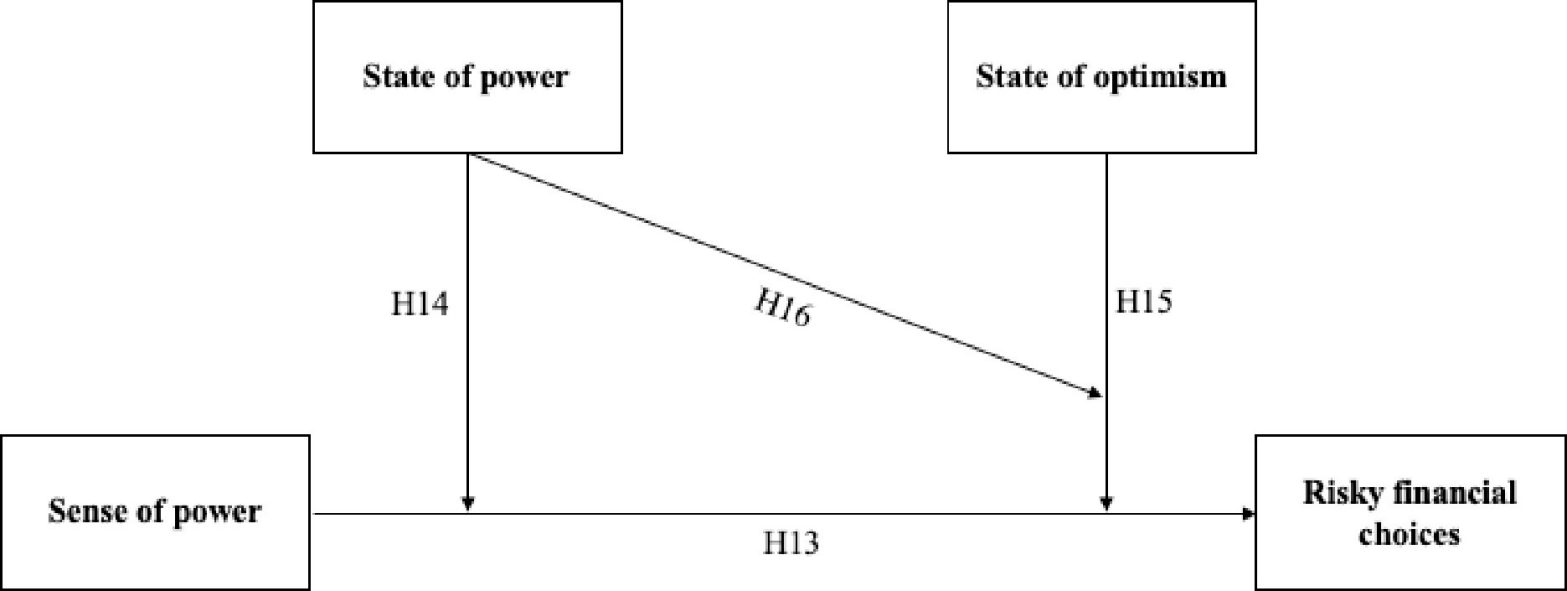
Study 3 framework and hypotheses.

Based on previous research, we expected that sense of power would be positively linked to risky financial choices (H13). Moreover, we expected that the states of optimism and power would moderate the positive relationship between sense of power and risky financial choices so that the relationship would be stronger in the state of power (as compared to the state of lack of power; H14) and in the state of optimism (as compared to state of pessimism; H15). We also hypothesized that the states of power and optimism would jointly moderate the positive relationship between sense of power and risky financial choices, so that the positive relationship would be the weakest in the state of lack of power and state of pessimism (H16). The research framework and hypotheses are shown in Fig 6 with the numbers of the hypotheses written above the arrows. The study was preregistered at: https://osf.io/ugzan/?view_only=dd620fe8e9c04e448fe32db86c32c4fd (DOI 10.17605/OSF.IO/UGZAN).

### 4.2. Methods

#### 4.2.1. Participants

Data were collected from 267 Polish working adults (140 female and 127 male; aged 19–64 years, *M* = 40.88 years, *SD* = 12.55). The Study was conducted using the same research panel as Study 1 and 2; the participants were also recruited analogously.

*4*.*2*.*1*.*1*. *Ethics approval and informed consent statements*. The Ethics Board at Faculty of Psychology, Warsaw University approved the study. All procedures performed in studies involving human participants were in accordance with the ethical standards of the institutional research committee and with the 1964 Helsinki Declaration and its later amendments or comparable ethical standards. Participants written informed consent was collected in the online Polish ARIADNA participant panel.

#### 4.2.2. Materials and procedure

**Sense of power, risky investment choices, and risky gambling choices** were measured using the same research tools as in Studies 1 and 2.

**Power as a state.** States of power and of lack of power were induced using the same research procedure as in Study 2.

**State of general optimism** was manipulated with a new research tool created by the authors for this study. In this tool, participants read about ambiguous events (e.g., receiving a parcel that was not ordered by the receiver) and wrote down either optimistic or pessimistic (depending on the condition) interpretations of the situations. Participants were first informed that in this task they will be asked to look at several events in an optimistic (or pessimistic) way. Next, they were provided with a passage depicting what it means to be optimistic (or pessimistic). Finally, they were asked to describe how they would interpret three ambiguous events if they were to think as an optimistic (or pessimistic) person. The exact instructions are presented in S1 File. The effectiveness of this manipulation was confirmed in the pilot study (a description of its results is presented in the S1 File).

**Fig 7:**
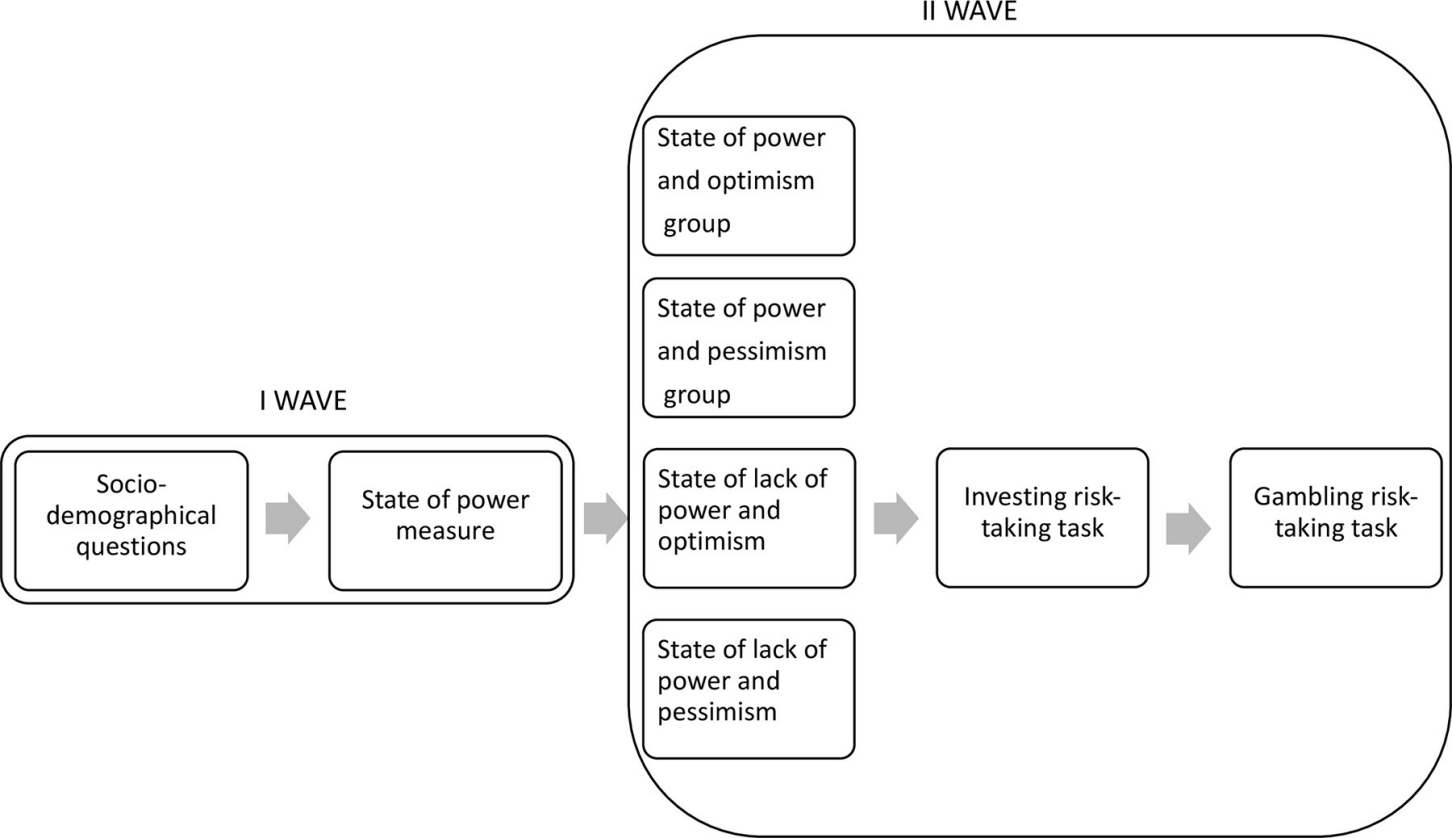
Flowchart of Study 3 procedure.

*4*.*2*.*2*.*1*. *Procedure*. The present study consisted of two waves. In the first wave, participants completed the Sense of Power questionnaire. Next, 6–7 days later, participants took part in the second wave of the study. At the beginning of the second wave, participants were randomly assigned to one of four experimental groups: (1) state of power and state of optimism (*n*_1_ = 69), (2) state of power and state of pessimism (*n*_2_ = 68), (3) state of lack of power and state of optimism (*n*_3_ = 67), and (4) state of lack of power and state of pessimism (*n*_*4*_ = 64). Then the participants took part in the state of power (or lack of power) experimental task followed by the state of optimism (or pessimism) experimental task. In the next step, they made investment and lottery choices. Finally, the participants were informed about the amount of additional extra points they will receive and were fully debriefed. The flowchart of the study is presented in Fig 7.

### 4.3. Results

#### 4.3.1. Descriptive statistics and correlations between variables

As shown in Table 3, all the expected relationships between variables were observed. SOP was positively albeit weakly related to risky investment choices (decision 1 and decision 2) and risky gambling choices. The relationship between the two investment choices was positive and strong. Moreover, the correlation between each investment decision and gambling decision was positive albeit weak.

Eight participants were excluded from all analyses related to the risky gambling choices due to their choice of dominated options in the lottery task.

**Table 3:** Means, standard deviations, and Pearson correlations between variables.

	*M*	*SD*	2	3	4
1. Sense of power	4.28	0.80	.21**	.27**	.33**
2. Risky investment choices–decision 1	45.31%	31.40%		.76**	.29**
3. Risky investment choices–decision 2	47.96%	31.60%			.30**
4. Risky gambling choices	5.13	2.86			

* *p* < .05

** *p* < .01, *Notes*: analyses for the variable 4 were conducted on a reduced sample (*n* = 259, 8 participants who made dominated choices in the tenth scenario in the lottery task were excluded from the analyses) and correlations related to the same model remained significant (*p* < .05) after Bonferroni correction for multiple comparisons; datasource: https://osf.io/ugzan/?view_only=dd620fe8e9c04e448fe32db86c32c4fd (DOI 10.17605/OSF.IO/UGZAN); research method: experimental.

#### 4.3.2. Joint moderation effect of states of power and optimism in the positive relationship between sense of power and risky financial choices

The PROCESS macro (Model 3) developed by Hayes [55] was used to determine the moderated moderation effect of state of power and state of optimism in the relationship between sense of power and risky financial choices (in both investing and gambling subdomains).

*4*.*3*.*2*.*1*. *Risky investment choices*. We conducted two hierarchical multiple regression analyses for risky investment choices with the first and second decisions as outcome variables. As the obtained results were very similar, we present the analyses only for decision 1, while the analyses for the second decision are given in the S1 File.

First, sense of power (SOP) was entered in step 1 of the model explaining risky investment choices, and the results showed that SOP was positively related to the dependent variable, which supported H13 (*F*(1,266) = 12.41, *p* < .001, Table 4). In step 2, in addition to SOP, state of power (StOP) and state of optimism (StOO) were also found to be significant positive predictors of propensity to take investment risks (*F*(3,264) = 19.89, *p* < .001, Table 4). Finally, in step 3, two- and three-way interactions between the independent variables were introduced into the model (*F*(7,260) = 11.62; *p* < .001). The effect of StOO remained significant. Moreover, all the analyzed interaction effects were significant (Table 4). In line with H14, the role of sense of power in explaining risky investment choices was stronger in the StOP condition (*ß* = .24, *p* = .005) than in the state of lack of power condition (*ß* = .21, *p* = .01). Moreover, as was expected in H15, the role of SOP in explaining risky investment choices was stronger in the StOO condition (*ß* = .37, *p* < .001) than in the state of pessimism condition (*ß* = .13, *p* = .15). The three-way interaction was significant. The results of its decomposition into two two-way interactions showed a significant interaction between StOO and SOP, but only under the state of lack of power condition (state of lack of power condition: effect: 24.51, *p* < .001; state of power condition: effect: -1,72; p = .77). Further analyzes showed that under the state of lack of power condition, the effect of SOP on risky investment choices was not observed when the state of pessimism was induced (effect: -2.92; LLCI = -11.92; ULCI = 6.07), while the effect was significant under the condition of StOO (effect: 21.59; LLCI = 13.11; ULCI = 30.06). Moreover, the effects under the conditions of states of power and pessimism (effect: 10.56; LLCI = 2.35; ULCI = 18.76) and when the state of power and optimism were induced (effect: 8.83; LLCI = 0.48; ULCI = 17.20) were significant. Thus, H16 was supported.

**Table 4:** Predictors of propensity to take investment risks (Study 3).

	Step 1	Step 2	Step 3
Sense of power (SOP)	8.27*** (2.35)	10.03*** (2.21)	-2.92 (4.57)
State of power (StOP)^1^		19.14*** (3.49)	-34.15 (27.75)
State of optimism (StOO)^2^		13.97*** (3.54)	-85.27*** (27.41)
SOP x StOP			13.48 * (6.18)
SOP x StOO			24.51*** (6.27)
StOP x StOO			100.61** (37.65)
SOP x StOP x StOO			-26.22** (8.65)
Intercept	9.95 (10.21)	-14.46 (10.28)	39.46 (20.41)
Observations	267	267	267
*R* ^*2*^	.05	.18	.24

Note: The table presents the *B* values with standard errors in parentheses. Sex is coded as 1 for female and 0 for male.

*p < .05

**p < .01

***p < .001

^1^StOP is coded as 1 for state of power and 0 for state of lack of power; ^2^StOO is coded as 1 for state of optimism and 0 for state of pessimism, datasource: https://osf.io/ugzan/?view_only=dd620fe8e9c04e448fe32db86c32c4fd; research method: experimental.

*4*.*3*.*2*.*2*. *Risky gambling choices*. Next, a hierarchical multiple regression analysis was conducted with risky gambling choices as an outcome variable. The results of step 1 of the analyses (Table 5) supported H13 and showed that SOP is positively related to risky gambling choices (*F*(1,257) = 30.53, *p* < .001, Table 5). Then, in step 2, StOP and StOO in addition to SOP were found to be significant positive predictors of propensity to take investment risks (*F*(3,255) = 21.00, *p* < .001, Table 5). In step 3, we introduced the two- and three-way interactions between the independent variables into the model (*F*(7,251) = 15.36; *p* < .001). The effects of StOO and StOP remained significant. All the analyzed interaction effects were significant (Table 5). The role of SOP in explaining risky gambling choices was stronger in StOP (*ß* = .52, *p* < .001) than in the state of lack of power (*ß* = .11, *p* = .24), which supports H14. Moreover, the role of SOP in explaining risky gambling choices was stronger in the StOO (*ß* = .45, *p* <. 001) than in the state of pessimism (*ß* = .30, *p* < .001), which is in line with our expectations (H15). The three-way interaction was significant. The results of its decomposition into two two-way interactions showed a significant interaction between StOO and SOP, but only under the state of lack of power condition (state of lack of power condition: effect: 2.03, *p* < .001; state of power condition: effect: -0.56; p = .28). Further analyzes showed that under the state of lack of power condition, the effect of SOP on risky gambling choices was not observed when the state of pessimism was induced (effect: -0.52; LLCI = -1.32; ULCI = 0.28; supporting H16), while the effect was significant under the condition of StOO (effect: 2.21; LLCI = 1.50; ULCI = 2.9). The effects under the conditions of states of power and pessimism (effect: 2.21; LLCI = 1.50; ULCI = 2.93) and when the states of power and optimism were induced (effect: 1.66; LLCI = 0.93; ULCI = 2.39) were significant.

**Table 5:** Predictors of propensity to take gambling risks (Study 3).

	Step 1	Step 2	Step 3
Sense of power (SOP)	1.15*** (0.21)	1.32*** (0.21)	-0.52 (0.41)
State of power (StOP)^1^		0.72* (0.32)	-10.41*** (2.44)
State of optimism (StOO)^2^		1.60*** (0.32)	-6.32** (2.42)
SOP x StOP			2.73 *** (0.55)
SOP x StOO			2.03*** (0.55)
StOP x StOO			9.66** (3.31)
SOP x StOP x StOO			-2.59** (0.76)
Intercept	0.29 (0.90)	-1.64 (0.93)	5.97** (1.81)
Observations	259	259	259
*R* ^*2*^	.10	.19	.30

Note: The table presents the *B* values with standard errors in parentheses. Sex is coded as 1 for female and 0 for male.

*p < .05

**p < .01

***p < .001

^1^StOP is coded as 1 for state of power and 0 for state of lack of power

^2^StOO is coded as 1 for state of optimism and 0 for state of pessimism, datasource: https://osf.io/ugzan/?view_only=dd620fe8e9c04e448fe32db86c32c4fd; research method: experimental.

## 5. Discussion

We conducted three studies examining the mechanisms underlying the effect of power on risky financial decisions. This work is based on previous studies showing that greater power, understood both as a trait and as a state, is linked to riskier financial choices [20, 21] and that people with a greater sense of power have a more optimistic perception of their own future and future in general and that an optimistic perception of risky behaviors mediates preferences for risk-taking [5, 36]. Their findings shed light on the mechanisms underlying the link between power and risk-taking. However, it was unclear whether the relationship between power and risky choices is mediated not only by a more optimistic perception of risk but also by optimism per se. Moreover, the moderating role of optimism needed to be investigated in the search for factors that mitigate and exaggerate the effects of power on risk-taking. In this research project, we aimed to further develop knowledge on the mediating and moderating roles of optimism in the relationship between power and risky financial choices and on the boundary conditions of this effect. We have considered power both as a trait and as a state and taken into account possible interactions between them. We have also measured and manipulated optimism. All the effects were observed in two domains of risky financial decisions–investment (two tasks) and gambling. It should be noted that in each study the obtained patterns of results were the same regardless of the dependent variable, which enhances our confidence in the results.

Study 1 aimed to analyze whether the relationship between sense of power and risky financial choices in investing and gambling domains is serially mediated by general optimism and financial risk perception. Its results confirmed the initial hypotheses and demonstrated that a greater sense of power translates into greater optimism, which, in turn, leads to lower investment risk perception and results in riskier financial choices. Participants made two investment choices and this pattern of results was observed for both of them. In the case of gambling choices, analogous serial mediation occurred.

The second study focused on situationally-induced power and powerlessness. It investigated the role of states of power and lack thereof in explaining people’s financial decisions, situational optimism, and perception of risk. The results indicated that people in a state of power differed from people lacking power in terms of their situational optimism and the riskiness of their financial choices (both investment and gambling). People having power were more optimistic, invested more, and made riskier gambling choices than those in control conditions and those who lacked power. Correspondingly, people in a state of lack of power were less optimistic, invested less, and made less risky choices than people in the remaining groups. Importantly, there were no differences between the conditions in terms of risk perception both in investment and gambling choices.

The third study tested the single and joint moderating effects of states of optimism and power in explaining the positive relationship between sense of power and risky investing and gambling choices. In line with expectations, the results of the study showed that the states of power and optimism jointly moderate the positive relationship between sense of power and risky financial choices. The significant interaction between state of optimism and sense of power was observed only under the state of lack of power condition. The lowest and non-significant effect of sense of power on risky investment choices was observed when the state of lack of power and the state of pessimism were induced, while the effect was significant when the state of optimism was induced. Under the state of power condition, the relationship between sense of power and risky investment choices was observed when either the state of optimism or the state of pessimism was induced. The same pattern of results was observed in the case of risky gambling choices.

### 5.1. Limitations

Although the obtained results seem promising, some limitations of this study should be acknowledged. For example, even though the investment and gambling tasks were incentivized, the amounts at risk were incomparably lower than the money at stake on the stock market. Nevertheless, taking into account that both decisions made in scenarios with hypothetical rewards and scenarios with real but low payoffs match decisions made with real and substantial ones [56, 57], we are confident in the obtained results. Next, the method used to manipulate participants’ optimism levels is novel and hadn’t been used in previous studies. Although this might rise a potential concern, pilot tests demonstrated the effectiveness of this experimental manipulation.

### 5.2. Further research

Understanding the mechanisms that enhance risk-taking propensities among the powerful is undoubtfully important but at the same time complex issue. Surely, optimism is not the only mediator that impacts this relationship. Further studies should seek other variables that would help us explore the link between power and propensity to take financial risks ad understand the mediators and boundary conditions of this relationship. Moreover, a natural direction for the current study, which employed hypothetical scenarios, is an analysis of data from individual investors, although it should be noted that the replication of results in such a setting would be possible for only some of the effects presented in this manuscript–specifically, the mediating ones.

### 5.3 Study contribution

Overall, the results of these three studies shed light on the way optimism mediates and moderates the effect of power on financial risk-taking and thus extends our theoretical knowledge of the consequences of power. It was demonstrated that when people feel more powerful, they perceive reality in a more optimistic manner. This in turn impacts their risk assessment and results in riskier financial decisions. In line with this logic, people with situational power are also more optimistic than people lacking power and make riskier financial choices. However, there are some boundary conditions for these effects. Specifically, even people with high levels of sense of power might find themselves in situations in which they are under the power of someone else, and the reverse is also true: people low in sense of power might gain situational power over others. Moreover, powerful people might sometimes find themselves in circumstances that temporarily make them pessimistic and, conversely, optimism may be induced in those lacking power. Our study shows that the relationship between sense of power and financial risk-taking is stronger when one is in a state of power (vs. lack of it) or state of optimism (vs. pessimism). Therefore, people high in sense of power might not be as likely as usual to take financial risks when they are under the power of somebody else and/or they have pessimistic thoughts induced. This work contributes also to the knowledge on financial risk-taking by showing the significance of factors that can modify the perception of riskiness of financial choices. Given the recent and impending economic crises [58, 59], such findings are of crucial importance. Expanding our understanding of the mechanisms underlying investment and gambling choices might provide insights for promoting more responsible financial behaviors. Furthermore, the present work is one of only a few attempts to explore the role of power in gambling and personal investment choices, and our findings extend the vast literature on risky financial choices by demonstrating how can they be modified by psychological states and traits. Finally, to the best of our knowledge, the joint impact of optimism and power has not been investigated previously.

## Supporting information

S1 File Additional information on materials used in the study and additional analyses. (DOCX)
