# Supplementary material for: The effect of state and trait power on financial risk-taking: The mediating and moderating roles of optimism
Source: PLoS One. 2022 Oct 31;17(10):e0276878. doi: 10.1371/journal.pone.0276878 (PMC9621424; doi:10.1371/journal.pone.0276878)
Supplement: S1 File — (DOCX) [file pone.0276878.s001.docx]

**The Effect of State and Trait Power on Financial Risk-taking: the Mediating and Moderating Roles of Optimism**

**Supporting information**

**Appendix 1**

**Additional Information on Materials Used in the Study**

**1. Polish Version of the Optimism Scale**

The Polish version of the tool was created by the authors of the present paper following the rules of Beaton et al. [1] for cultural adaptation of psychometric questionnaires. The adaptation procedure consisted of five steps: 1) translation of the English version of the scale into Polish by three professional translators; 2) synthesis of the three translations into one Polish version by an expert holding a Ph.D. degree in psychology; 3) blind back-translation of the Polish version into English by three independent professional translators; 4) synthesis of the back-translations into one version by an expert (Ph.D. in psychology); and 5) expert review (2 experts with Ph.Ds in psychology, 1 with an MA in psychology) comparing the synthesized back-translation with the original version and finalizing the items of the scale. This procedure generated the final version of the scale, which was submitted to preliminary testing. The Polish Optimism scale is presented below. In order to confirm the factor structure of the Polish version of the questionnaire, Confirmatory Factor Analysis (CFA) was conducted (*N* = 328). Results showed that a one factor structure of the tool exhibits good model fit, 𝜒^2^=129.647, *df* = 27, *p* < 0.001, *CFI* = 0.922, *RMSEA* = 0.108 (90% *CI* = 0.089 to 0.127), *SRMSR* = 0.046. Although the RMSEA is too high, some scholars claim that this is not a reliable indicator of fit for a CFA with few degrees of freedom [2], therefore the remaining indicators can be interpreted as satisfactory. The reliability of the Polish version of the scale was good in our sample (Cronbach's $\alpha$ = 0.88).

**Polish Version of the Optimism Scale**

Przeczytaj poniższe zdania i oceń na ile zgadzasz się z każdym z nich, biorąc pod uwagę to, **co zazwyczaj myślisz i czujesz** używając skali:

*1 – zdecydowanie się nie zgadzam*

*2 - trochę się nie zgadzam*

*3 – ani się zgadzam ani nie zgadzam*

*4 – trochę się zgadzam*

*5 – zdecydowanie się zgadzam*

OP1. Wierzę, że zrealizuję moje główne życiowe cele.

OP2. Kiedy myślę o przyszłości, mam pozytywne nastawienie.

OP3. Przydarza mi się więcej dobrych rzeczy niż złych.

OP4. Myślę, że wszystko pójdzie źle.

OP5. Widzę każde wyzwanie jako szansę na sukces.

OP6. Dostrzegam pozytywne aspekty sytuacji, nawet jeśli sprawy idą źle.

OP7. Dostrzegam pozytywne strony różnych sytuacji.

OP8. Mam pewność, że pokonam problemy.

0P9. Ze spokojem patrzę na swoją przyszłość.

**2. Situationally Induced General Optimism** – **Manipulation Tool**

**Optimistic Condition**

For the purposes of this assignment, we would like to ask you to try to look at some of the situations described below through the eyes of an optimist, regardless of whether you would normally describe yourself this way or not.

Being optimistic about a situation means expecting it to turn out well. The optimist sees the world positively, through rose-colored glasses, according to the rule: if something can go well, it will go well. For an optimist, the glass is always half full, and he sees the bright sides in every situation.

**1. What would you think about the following situation as an optimist? You hear through the intercom the voice of the postman saying, "I have a package for you", even though you have not ordered anything.**

*Write down below what comes to your mind.*

**2. What would you think about the following situation as an optimist? A holiday resort employee called you to say that you have been mistakenly booked into the wrong holiday home and therefore they must transfer your reservation to another resort located nearby.**

*Write down below what comes to your mind.*

**3. What would you think about the following situation as an optimist? You have received a text message from a friend telling you that you need to talk urgently because he has an important message for you.**

*Write down below what comes to your mind.*

**Pessimistic Condition**

For the purposes of this assignment, we would like to ask you to try to look at some of the situations described below through the eyes of a pessimist, regardless of whether you would normally describe yourself this way or not.

Being a pessimist about a situation means expecting it to turn out poorly. A pessimist views the world negatively, expecting the worst possible scenarios of events, according to the principle: if something can go wrong, it will go wrong. For a pessimist, the glass is always half-empty, and in every situation, he sees the darker sides.

**1. What would you think about the following situation as a pessimist? You hear through the intercom the voice of the postman saying, "I have a package for you", even though you have not ordered anything.**

*Write down below what comes to your mind.*

**2. What would you think about the following situation as a pessimist? A holiday resort employee called you to say that you have been mistakenly booked into the wrong holiday home and therefore they must transfer your reservation to another resort located nearby.**

*Write down below what comes to your mind.*

**3. What would you think about the following situation as a pessimist? You have received a text message from a friend telling you that you need to talk urgently because he has an important message for you.**

*Write down below what comes to your mind.*

**3. Situationally Induced General Optimism** – **Results of the Pilot Study**

In total, 95 participants took part in the pilot study, 85 women and 10 men, aged 18 to 45 (*M* = 26.7, *SD* = 5.55). Participants were randomly assigned to one of the experimental conditions (optimism, *N* = 45; pessimism, *N* = 50). First, participants were given a task that aimed to situationally induce optimism or pessimism (the experimental manipulation used in later studies). They were asked to interpret three ambiguous situations in an optimistic or pessimistic way (depending on the condition). They were to write down their interpretations in a few words. Next, they completed the Optimism Scale [3], in its Polish version with modified instruction, which asks about people's current feelings to capture their situational level of general optimism. The results showed significant differences between the groups in terms of situational levels of general optimism (*t*(93) = 2.257, *p* < .05, *Cohen’s d* = .464). The participants in the optimism experimental group had greater situational levels of general optimism (*M* = 3.77, *SD* = 0.79) than those in the pessimism group (*M* = 3.42, *SD* = 0.73). The results of the study confirmed the effectiveness of the manipulation: the manipulation task influences people’s situational optimism in the expected direction.

**4. Power Manipulation**

**Experimental Condition: Power**

Last week, research concerning creativity was conducted among our panelists. Participants were asked to write short stories comprising three comprehensible sentences in which they had to use three provided words in such a way that it was difficult to guess which word had been provided. In other words, the words provided should have been concealed as much as possible.

The best stories will be used in future studies and rewarded with extra points exchangeable for rewards offered by the platform running the panel.

Today your task is to evaluate the stories and to decide whether to reward their authors with extra points and, if so, how many points should be awarded. You will also decide whether a particular story will be used in further studies.

Read each of the stories below and answer the questions provided.

In all the stories the same three words have been concealed: plane, pencil case, and rope (either plural or singular).

Story 1. (author number 217)

This September Krystian is going to a new school, which makes him very excited, and motivates him to prepare everything in advance. He has a new backpack with a picture of a plane on it and a colorful pencil case full of colorful felt tip pens and crayons. Krystian can’t wait for the first day of school because the jump rope contest will take place and Krystian is strong and thinks that he will win.

1. Evaluate the task performance (very poor/excellent)
2. Should this story be used in further research? (yes/no)
3. Should the author of this story be rewarded? The prize is in Polish zloty and the money will be converted to points exchangeable for rewards. Using the scale provided below, indicate the amount of the prize to be awarded to the author of this story or choose 0 if in your opinion the author should not receive a reward. (PLN 0 – PLN 10)

Story 2. (author number 1102)

Janek lives near Okęcie Airport and has always dreamt about flying by plane. Since he was a child he has always chosen clothes, toys, and school accessories, such as backpacks or pencil cases, with pictures of planes. Recently, his dream was supposed to come true – he was to fly by plane for the first time, but he tripped over a rope lying on the ground, broke his leg, and had to postpone his journey.

1. Evaluate the task performance (very poor/excellent)
2. Should this story be used in further research? (yes/no)
3. Should the author of this story be rewarded? The prize is in Polish zloty and the money will be converted to points exchangeable for rewards. Using the scale provided below, indicate the amount of the prize to be rewarded to the author of this story or choose 0 if in your opinion the author should not receive a reward. (PLN 0 – PLN 10)

Story 3. (author number 754)

Maria was hanging the laundry on ropes stretched across the front yard. Suddenly she heard that her daughter was shouting something at her, but she did not understand what it was because the plane flying overhead completely drowned out what the girl was trying to say. After a while, everything was clear, Maria fell forcefully to the ground, stumbling over her daughter's schoolbag and pencil case.

1. Evaluate the task performance (very poor/excellent)
2. Should this story be used in further research? (yes/no)
3. Should the author of this story be awarded? The prize is in Polish zloty and the money will be converted to points exchangeable for rewards. Using the scale provided below, indicate the amount of the prize to be rewarded to the author of this story or choose 0 if in your opinion the author should not receive a reward. (PLN 0 – PLN 10)

**Experimental Condition: Lack of Power**

Your task is to write a short story comprising three comprehensible sentences using three provided words in such a way that it is difficult to guess which word has been provided. In other words, the words provided should be concealed as much as possible.

The best stories will be used in future studies and rewarded with extra points exchangeable for rewards offered by the platform running the panel.

Another panelist will evaluate the stories and decide whether you will be rewarded extra points and, if so, how many points should be awarded.

This person will read your story and answer the following questions:

1. Evaluate the task performance (very poor/excellent)
2. Should this story be used in further research? (yes/no)
3. Should the author of this story be rewarded? The prize is in Polish zloty and the money will be converted to points exchangeable for rewards. Using the scale provided below, indicate the amount of the prize to be awarded to the author of this story or choose 0 if in your opinion the author should not receive a reward. (PLN 0 – PLN 10)

To better understand this task, you will see three stories written by other panelists.

In all the stories the same three words have been concealed: plane, pencil case, and rope (either plural or singular).

Story 1. (author number 217)

This September Krystian is going to a new school, which makes him very excited, and motivates him to prepare everything in advance. He has a new backpack with a picture of a plane on it and a colorful pencil case full of colorful felt tip pens and crayons. Krystian can’t wait for the first day of school because the jump rope contest will take place and Krystian is strong and thinks that he will win.

Story 2. (author number 1102)

Janek lives near Okęcie Airport and has always dreamt about flying by plane. Since he was a child he has always chosen clothes, toys, and school accessories, such as backpacks or pencil cases with pictures of planes. Recently, his dream was supposed to come true – he was to fly by plane for the first time, but he tripped over a rope lying on the ground, broke his leg, and had to postpone his journey.

Story 3. (author number 754)

Maria was hanging the laundry on ropes stretched across the front yard. Suddenly she heard that her daughter was shouting something at her, but she did not understand what it was because the plane flying overhead completely drowned out what the girl was trying to say. After a while, everything was clear, Maria fell forcefully to the ground, stumbling over her daughter's schoolbag and pencil case.

Now write your own story using the following three words: lamp, penguin, and tree (singular or plural).

**Experimental Condition: Control**

Your task is to write a short story comprising three comprehensible sentences using three provided words in such a way that it is difficult to guess which word has been provided. In other words, the words provided should be concealed as much as possible.

Your story will be added to our story database.

To better understand this task, you will see three stories written by other panelists.

In all the stories the same three words have been concealed: plane, pencil case, and rope (either plural or singular).

Story 1. (author number 217)

This September Krystian is going to a new school, which makes him very excited, and motivates him to prepare everything in advance. He has a new backpack with a picture of a plane on it and a colorful pencil case full of colorful felt tip pens and crayons. Krystian can’t wait for the first day of school because the jump rope contest will take place and Krystian is strong and thinks that he will win.

Story 2. (author number 1102)

Janek lives near Okęcie Airport and has always dreamt about flying by plane. Since he was a child he has always chosen clothes, toys, and school accessories, such as backpacks or pencil cases with pictures of planes. Recently, his dream was supposed to come true – he was to fly by plane for the first time, but he tripped over a rope lying on the ground, broke his leg, and had to postpone his journey.

Story 3. (author number 754)

Maria was hanging the laundry on ropes stretched across the front yard. Suddenly she heard that her daughter was shouting something at her, but she did not understand what it was because the plane flying overhead completely drowned out what the girl was trying to say. After a while, everything was clear, Maria fell forcefully to the ground, stumbling over her daughter's schoolbag and pencil case.

Now write your own story using the following three words: lamp, penguin, and tree (singular or plural).

**5. Holt and Laury Lottery Task (with Polish Currency) with an Explanation of the Rules for Awarding Additional Points**

In the next task, you will make ten decisions. Your choices will impact the number of extra points you will be awarded for participation in this study.

Each decision is a choice between “Option A” and “Option B”.

Please look at the table below to understand this task. Look at “Decision 1”.

**Option A** pays:

- PLN 2.00 if the throw of a ten-sided die is 1

or

- PLN 1.60 if the throw is one of the 9 remaining sides (2–9)

**Option B** pays:

- PLN 3.85 if the throw of a ten-sided die is 1

or

- PLN 0.10 if the throw is one of the 9 remaining sides (2–9)

The other decisions are similar, except that in each subsequent decision the chances of the higher payoff increase both in Option A and Option B.

After you make all ten decisions, the computer will draw one of them and this decision will be used to determine the number of extra points you will receive for this part of the survey. Next, one side of a ten-sided die will be thrown, and it will determine the number of extra points you will get at the end of the study.

Please mark your choice (Option A or Option B) next to each of the following 10 decisions.

|  | Option A | Option B |
| --- | --- | --- |
| Decision 1 | 1/10 gain of PLN 2; 9/10 gain of PLN 1.60 | 1/10 gain of PLN 3.85; 9/10 gain of PLN 0.10 |
| Decision 2 | 2/10 gain of PLN 2; 8/10 gain of PLN 1.60 | 2/10 gain of PLN 3.85; 8/10 gain of PLN 0.10 |
| Decision 3 | 3/10 gain of PLN 2; 7/10 gain of PLN 1.60 | 3/10 gain of PLN 3.85; 7/10 gain of PLN 0.10 |
| Decision 4 | 4/10 gain of PLN 2; 6/10 gain of PLN 1.60 | 4/10 gain of PLN 3.85; 6/10 gain of PLN 0.10 |
| Decision 5 | 5/10 gain of PLN 2; 5/10 gain of PLN 1.60 | 5/10 gain of PLN 3.85; 5/10 gain of PLN 0.10 |
| Decision 6 | 6/10 gain of PLN 2; 4/10 gain of PLN 1.60 | 6/10 gain of PLN 3.85; 4/10 gain of PLN 0.10 |
| Decision 7 | 7/10 gain of PLN 2; 3/10 gain of PLN 1.60 | 7/10 gain of PLN 3.85; 3/10 gain of PLN 0.10 |
| Decision 8 | 8/10 gain of PLN 2; 2/10 gain of PLN 1.60 | 8/10 gain of PLN 3.85; 2/10 gain of PLN 0.10 |
| Decision 9 | 9/10 gain of PLN 2; 1/10 gain of PLN 1.60 | 9/10 gain of PLN 3.85; 1/10 gain of PLN 0.10 |
| Decision 10 | 10/10 gain of PLN 2; 0/10 gain of PLN 1.60 | 10/10 gain of PLN 3.85; 0/10 gain of PLN 0.10 |

1. **The riskiness of investment choices**

In the next step, you will see two charts depicting changes in the price of hypothetical Stocks X and Y over the last 10 years. The charts do not present the results of any existing company, they were generated by a computer following certain criteria.

After that, you will be asked to make two financial decisions, which will likely affect the number of extra points you will receive after the study.

You will have hypothetical PLN 10 000 of savings, that you will be able to divide between an interest-free bank account and Stocks X or Stocks Y. The decision is made over a one-year period and the inflation rate is always 0.

At the end of the study, you will receive information regarding the price of Stock X and Stock Y after a year and your current financial outcome. The Stock price after a year will be computed based on the same criteria that were assumed while generating the historical Stock prices presented in the chart.

Next, we will compute your total financial outcome and exchange it into Ariadna ^[[1]](#footnote-1)^ points that you will be able to use later in the Ariadna shop following the rule: PLN 2500 = 0.5 points.

If you put all the money into the bank account, you will get 4 extra points for this task. If you decide to invest all the money, your final score will depend on the Stock prices and might be higher or lower than the result achieved when all the money is assigned to the bank account.

/next screen/

A chart depicting the historical prices of Stocks X over the last 10 years is presented below. Please take a look at it.

The price of one Stock X equals PLN 6

Please divide PLN 10 000 of savings between Stocks X and an interest-free bank account Please assume that the inflation rate is 0.

.......... to an interest-free bank account

.......... Stocks X

/next screen/

A chart depicting the historical prices of Stocks y over the last 10 years is presented below. Please take a look at it.

The price of one Stock Y equals PLN 5.90

Please divide PLN 10 000 of savings between Stocks Y and an interest-free bank account Please assume that the inflation rate is 0.

.......... to an interest-free bank account

.......... Stocks Y

**Appendix 2**

**Additional Analises**

**1. Study 1**

**Risky Investment Choices – Decision 2**

As can be seen in Figure 2, in line with our predictions, sense of power was positively related to optimism and risky investment choices (which supports H1 and H2) and negatively related to risk perception (which supports H3). Optimism was positively related to risky financial choices and negatively related to risk perception (supporting H4 and H5). Moreover, in line with H6, risk perception was negatively linked to risky financial choices.


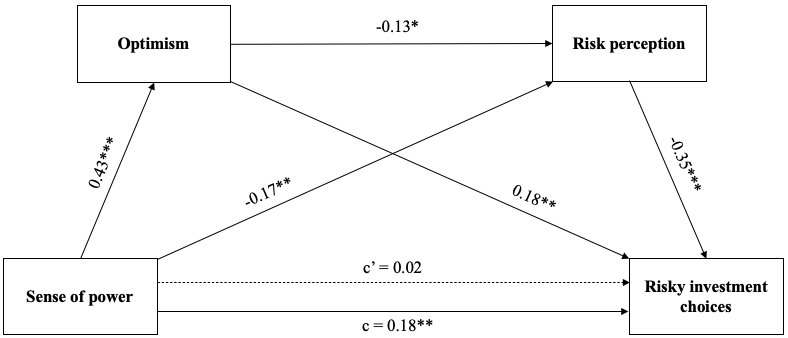


**Figure 1.** Serial multiple mediation model explaining risky investment choices. For the path directly linking sense of power and risky choices, the direct effect is shown above the upper arrow and the total effect (the effect without controlling for the mediator variable) is shown below the lower arrow. The statistics represent standardized effects. Solid arrows represent significant effects (****p* < .001, ***p* < .01, **p* < .05**; c – total effect, c’ – direct effect).

The overall regression model explaining risky investment choices with sense of power, optimism, and risk perception introduced as independent variables was significant (*R^2^*=.19; *F*(3,324) = 25.42*; p<*.001). As predicted in H1, the total effect (c) of sense of power on risky investment choices was positive and significant. The direct effect (c’) of sense of power in risky investment choices was not significant in the serial mediation model, while the total indirect effect was significant (c-c’= .16; BootSE = .03; 95% CI = .09 to .23), which indicates full mediation. Further analyses showed that the total indirect effect of sense of power on risky investment choices can be decomposed into three different significant partial indirect effects. The first was the effect via optimism. Sense of power was related to increased optimism, which translated into risky investment choices (partial effect 1, indirect effect = 0.08; BootSE=.03; 95% CI = .03 to .13; power: 0.91). The second partial indirect effect was via risk perception – sense of power was related to lowered risk perception which in turn was related to riskier investment choices (partial effect 2, indirect effect = 0.06; BootSE=.03; 95% CI = .01 to .12; power: 0.82). Finally, the serial mediation effect was also observed. Sense of power translated into greater optimism, which led to lower investment risk perception, which, in turn, led to riskier investment choices (partial effect 3, indirect effect = 0.02; BootSE=.01; 95% CI = .001 to .04; power: 0.60). Partial indirect effects 1 and 2 played a similar role, while the role of partial indirect effect 3 was smaller.

**2. Study 3**

**Joint Moderation Effect of States of Power and Optimism in the Positive Relationship Between Sense of Power and Risky Investment Choices – Investment Decision 2**

The PROCESS macro (Model 3) developed by Hayes (2013) was used to determine the moderated moderation effect of state of power and state of optimism in the relationship between sense of power and risky investment choices

We conducted a hierarchical multiple regression analysis with risky investment choice in decision 2 as the outcome variable.

We first entered sense of power (SOP) in step 1 of the model explaining risky investment choices, and the results showed that SOP was positively related to the dependent variable, which supported H13 (*F*(1,266) = 20.12, *p* < .001, Table 6). In step 2, next to the SOP, state of power (StOP) and state of optimism (StOO) were also found to be significant positive predictors of propensity to take investment risks (*F*(3,264) = 26.04, *p* < .001, Table 6). Lastly, in step 3, the two- and three-way interactions between the independent variables were introduced into the model (*F*(7,260) = 15.12; *p* < .001). The effect of state of optimism remained significant. Moreover, all the analyzed interaction effects were significant (Table 6). In line with H14, the role of SOP in explaining risky investment choices was stronger in StOP (*ß* = .30, *p* = .005) than in the state of lack of power (*ß* = .28, *p* < .001). Moreover, as was expected in H15, the role of sense of power in explaining risky investment choices was stronger in StOO (*ß* = .44, *p* < .001) than in the state of pessimism (*ß* = .15, *p* = .10). The three-way interaction was significant and showed that the lowest (and also non-significant) effect of SOP on risky investment choices was observed when the states of lack of power and pessimism were induced (effect: -1.58; LLCI = -10.33; ULCI = 7.16), while the effect was the strongest under the condition of state of lack of power and state of optimism (effect: 26.64; LLCI = 2.49; ULCI = 18.75). The effects under condition of states of power and pessimism (effect: 10.95; LLCI = 2.98; ULCI = 18.93) and when the states of power and optimism were induced (effect: 10.62; LLCI = 2.49; ULCI = 18.75) were significant. Thus, H16 was supported.

**Table 1.** Predictors of propensity to take investment risks (Study 2)

|  | Step 1 | Step 2 | Step 3 |
| --- | --- | --- | --- |
| Sense of power (SOP)  State of power (StOP)^1^  State of optimism (StOO)^2.^  SOP x StOP  SOP x StOO  StOP x StOO  SOP x StOP x StOO  Intercept | 10.45***  (2.33)  3.28  (10.14) | 12.08***  (2.17)  22.04***  (3.41)  12.43***  (3.47)  -21.24  (10.07) | -1.58  (4.44)  -30.69  (29.97)  -106.13***  (26.65)  12.54 *  (6.01)  28.22***  (6.10)  117.17**  (36.60)  -28.55**  (8.41)  37.65  (19.84) |
| Observations  *R^2^* | 267  .07 | 267  .23 | 267  .29 |

Note: The table presents the *B* values with standard errors in parentheses. Sex is coded as 1 for female and 0 for male. **p* <.05; ***p* <.01; ****p* <.001; ^1^StOP is coded as 1 for state of power and 0 for state of lack of power; ^2^StOO is coded as 1 for state of optimism and 0 for state of pessimism

**Bibliography**

1. Beaton DE, Bombardier C, Guillemin F, Ferraz MB. Guidelines for the process of cross-cultural adaptation of self-report measures. Spine. 2000. doi:10.1097/00007632-200012150-00014

2. Kenny DA, Kaniskan B, McCoach DB. The Performance of RMSEA in Models With Small Degrees of Freedom. Sociological Methods and Research. 2015;44. doi:10.1177/0049124114543236

3. Coelho GLH, Vilar R, Hanel PHP, Monteiro RP, Ribeiro MGC, Gouveia V v. Optimism scale: Evidence of psychometric validity in two countries and correlations with personality. Personality and Individual Differences. 2018;134. doi:10.1016/j.paid.2018.06.030

1. The study was run on a platform called Ariadna. The platform offers points for participation in various studies that can be subsequently exchanged in the Ariadna shop for gifts of participant’s choice. [↑](#footnote-ref-1)
